# Targeting of T/Tn Antigens with a Plant Lectin to Kill Human Leukemia Cells by Photochemotherapy

**DOI:** 10.1371/journal.pone.0023315

**Published:** 2011-08-17

**Authors:** Guillaume Poiroux, Marguerite Pitié, Raphaël Culerrier, Elodie Lafont, Bruno Ségui, Els J. M. Van Damme, Willy J. Peumans, Jean Bernadou, Thierry Levade, Pierre Rougé, Annick Barre, Hervé Benoist

**Affiliations:** 1 Institut National de la Santé et de la Recherche Médicale UMR 1037, Equipe 4, Centre de Recherches en Cancérologie de Toulouse, CHU Rangueil, BP84225, 31432 Toulouse, France; 2 Université de Toulouse, Faculté des Sciences Pharmaceutiques, 35 chemin des Maraîchers, 31062 Toulouse, France; 3 Université de Toulouse, UMR UPS-CNRS 5546, 24 Chemin de Borde Rouge, 31326 Castanet-Tolosan, France; 4 Centre National de la Recherhce Scientifique, Laboratoire de Chimie de Coordination, 205 route de Narbonne, F-31077, Toulouse, France; 5 Department of Molecular Biotechnology, Laboratory of Biochemistry and Glycobiology, Ghent University, Coupure links 653, B-9000 Ghent, Belgium; Institut Pasteur, France

## Abstract

Photochemotherapy is used both for solid tumors and in extracorporeal treatment of various hematologic disorders. Nevertheless, its development in oncology remains limited, because of the low selectivity of photosensitizers (PS) towards human tumor cells. To enhance PS efficiency, we recently covalently linked a porphyrin (TrMPyP) to a plant lectin (Morniga G), known to recognize with high affinity tumor-associated T and Tn antigens. The conjugation allowed a quick uptake of PS by Tn-positive Jurkat leukemia cells and efficient PS-induced phototoxicity. The present study was performed: *(i)* to evaluate the targeting potential of the conjugate towards tumor and normal cells and its phototoxicity on various leukemia cells, *(ii)* to investigate the mechanism of conjugate-mediated cell death. The conjugate: (*i*) strongly increased (×1000) the PS phototoxicity towards leukemic Jurkat T cells through an O-glycan-dependent process; (*ii*) specifically purged tumor cells from a 1∶1 mixture of Jurkat leukemia (Tn-positive) and healthy (Tn-negative) lymphocytes, preserving the activation potential of healthy lymphocytes; (*iii*) was effective against various leukemic cell lines with distinct phenotypes, as well as fresh human primary acute and chronic lymphoid leukemia cells; (*iv*) induced mostly a caspase-independent cell death, which might be an advantage as tumor cells often resist caspase-dependent cell death. Altogether, the present observations suggest that conjugation with plant lectins can allow targeting of photosensitizers towards aberrant glycosylation of tumor cells, *e.g.* to purge leukemia cells from blood and to preserve the normal leukocytes in extracorporeal photochemotherapy.

## Introduction

Extracorporeal photochemotherapy (ECP) is reported to be effective for a wide variety of hematologic disorders, such as graft-versus-host disease (GVH) or T cell lymphoma [1]. After cytapheresis, mononuclear cells are treated with a photosensitizer (PS), irradiated by ultraviolet-A (UV-A), and then reinfused to the patient. Alternatively, photodynamic therapy (PDT) uses other PS molecules and visible light to kill solid tumors [2]. Although the molecular mechanisms induced by ECP and PDT partially differ, both photochemotherapy (PCT) methods may be used to decrease the tumor mass. In contrast to surgery, radiotherapy and chemotherapy, PCT is known to stimulate the anti-cancer immunity [3], [4], [5]. In addition, PCT can be effective against chemo- and radio-resistant tumors [6].

So far, the relative poor selectivity of PS for tumor cells has remained a major drawback for the development of PCT applications. For instance, ECP efficacy could be increased through targeting of PS to tumor cells, therefore sparing healthy leukocytes, which would be reinfused to the patient with dead malignant leukocytes. With respect to solid tumors, therapeutic selectivity of PDT is achieved from the relative preferential localization of PS in the tumor tissue, owing to its physicochemical properties (*e.g.*, hydrophobicity), and from the control of irradiated areas (*e.g.*, in dermatologic application). However, in complex anatomical sites, such as the thoracic or abdominal cavities, confined irradiation is more difficult and treatment may injure normal tissue. Consequently, targeting of PS by increasing drug concentration in tumor tissues must be sought to enhance PCT efficacy.

Alterations of protein and lipid glycosylation at the cell surface are consistent features of cancers. In tumors, aberrant glycosylation is responsible for incomplete synthesis of the carbohydate chains, allowing higher expression of precursor carbohydrate moieties, such as the “T/Tn antigens” [*i.e.* CD176/CD175 antigens or (Galβ1-3GalNAcα1-O-Ser/Thr)/(GalNAcα1-O-Ser/Thr) epitopes] [7], [8]. These glycopeptide antigens are associated with many cancers and represent attractive candidates, among the tumor-associated carbohydrate antigens, for the development of anticancer immune stimulation [9] and drug targeting strategies [10]. Thus, the preparation of anti-Tn antibodies has been reported, but their evaluation in treatment or imaging of tumors is generally inconsistent [10], [11].

Along with antibodies, some plant lectins represent targeting-vectors because of their capacity to specifically recognize sugars and to distinguish subtle alterations in glycans on the cell surface [12], [13], [14]. Thus, Morniga G (MorG), a hetero-tetrameric (α4β4) lectin from *Morus nigra* was described as T/Tn (CD176/CD175) antigen-specific in cell-free systems [15]. Consequently, this lectin can be proposed for drug targeting towards tumor cells strongly expressing T and/or Tn antigens. Recently, we have demonstrated that MorG can be specifically bound and taken-up by a Tn-positive (Jurkat) lymphoid leukemia cell line [16]. For the first time, the lectin was covalently conjugated to TrisMPyP (TrMPyP), a cationic and hydrophilic porphyrin known to be white-light activatable [17]. The TrMPyP-MorG conjugate was molecularly characterized. The conjugate (with a 1∶1 PS∶lectin ratio) was bound and quickly (5 min) taken-up by Jurkat cells. Preliminary data indicate that the conjugate could trigger greater than 90% phototoxicity on leukemic Jurkat cells at 10 nM concentration [16]. In the present work, the role of O-glycosylation recognition for the conjugate-induced phototoxicity was studied. Because of the presence of CD175 and CD176 antigens on leukemia cells and their absence on normal mature hematopoietic cells [7], the conjugate-induced phototoxicity was comparatively evaluated towards Jurkat leukemia T cells (CD175-positive) and healthy T lymphocytes (CD175-negative). The phototoxicity of this new conjugate was also tested against various human leukemia cell lines and fresh primary leukemia cells from patients. Finally, the mechanism of conjugate-mediated photoxicity was investigated.

## Methods

### Cells and reagents

Jurkat T, Molt 4, CEM, HuT78, K562, KG1, KG1a, HL60 and U937 leukemia cell lines (from ATCC), SKW6.4 cells (EBV-transformed B lymphoid cell line, from ATCC) and ERG cells (EBV-transformed B lymphoid cell line from our laboratory), FADD-deficient Jurkat cells (from Dr. J. Blenis, Boston, MA, USA), caspase 9-deficient Jurkat cells (from Dr. K. Shulze-Osthoff, Düsseldorf, Germany), Caspase 8- and 10-doubly deficient Jurkat cells (from our laboratory [18]) were cultured in RPMI containing 10% FCS (Gibco, Cergy-Pontoise, France). Primary acute lymphoid leukemia (ALL, pre-B phenotype for ALL1, ALL2, and ALL3 and bi T/B phenotype for ALL4, T phenotype for ALL5) samples were obtained from Tumorothèque de Midi-Pyrénées (HIMIP, Inserm-U563, Purpan Hospital, Toulouse). Primary chronic lymphoid leukemia (CLL, B phenotypes) and healthy samples were obtained from Hematology department (Rangueil Hospital, Toulouse). PBMC from healthy donors and leukemic patients were separated using Ficoll-Paque Plus (Amersham Biosciences, Piscataway, NJ, USA) density gradient. Normal T lymphocytes were separated from PBMC using the Rosette Sep human T cell enrichment cocktail (StemCell Technologies, Grenoble, France) [14].

Morniga G (MorG) was purified from the bark of black mulberry tree (*Morus nigra*) and labelled with FITC (Acros Organics, Fisher Scientific, Illkirch, France) as previously described [14]. Anti-CD175 (anti-Tn) mAb and secondary PE-conjugated anti-mouse goat polyclonal antibodies were from Acris Antibodies GmbH (Herford, Germany). z-VAD(OMe)-fmk was purchased from Bachem (Voisins-Le-Bretonneux, France). Mannan, Lactoferrin and Bovine Submaxillary Mucin (BSM) were from Sigma-Aldrich (L'Isle d'Abeau Chesnes, France).

### Ethics statement

Fresh and thawed samples from ALL patients have been obtained after informed consent and stored at the HIMIP collection. According to the French law, HIMIP collection has been declared to the Ministry of Higher Education and Research (DC 2008-307 collection 1) and obtained a transfer agreement (AC 2008-129) after approbation by the “Comité de Protection des Personnes Sud-Ouest et Outremer II” (ethical committee). Clinical and biological annotations of the samples have been declared to the CNIL (Comité National Informatique et Libertés ie Data processing and Liberties National Committee).

Blood samples from healthy donors and chronic lymphocytic leukemia (CLL) patients were collected by Hematology department (Rangueil Hospital, Toulouse), after patients' written informed consent in accordance with the Declaration of Helsinski. For CLL, the samples were waste material took in diagnostic goal.

### Preparation and characterization of TrMPyP-MorG conjugates

TrisMPyP [5-(4-(5-carboxy-1-butoxy)-phenyl)-10,15,20-tris(4-N-methyl)-pyridiniumyl-porphyrin] was synthesized and its N-hydroxysuccinimidyl activated ester was prepared as described previously [19], then TrMPyP-MorG conjugates were prepared and characterized using SDS-PAGE analysis, MALDI-TOF mass spectrometry analysis and spectrophotometry analysis [16]. A TrMPyP-MorG conjugate with a mean of 1 PS per MorG lectin molecule and without alteration of the sugar recognition specificity of MorG was selected and used in the present experiments.

### Photodynamic treatment and cell death evaluation

Cells (10^6^/mL) were exposed to MorG, TrMPyP or TrMPyP-MorG, with or without inhibitory sugar or glycoprotein solutions for 15 min at 37°C, then washed. Because TrMPyP is white-light activatable, cells were irradiated with a bank of four white fluorescent tubes (Philips master TL-D 18W/840) for 7.5 min (1.7 J/cm^2^ light dose). Cell viability was evaluated using the MTT reduction method (Euromedex, Souffelweyersheim, France) or by flow cytometry using Annexin V-FITC and/or propidium iodide staining (AbCys, Paris, France). The formation of acidic vesicular organelles (AVOs, including autolysosomes) was evaluated by flow cytometry using the pH-sensitive fluorescent dye acridine orange (AO; Sigma). In viable cells, the concentration of AO in lysosomes results in high red fluorescence. Starvation-induced autophagy triggers an increase of red fluorescence of cells corresponding to an increased number of AVOs [20]. Cell morphology was analyzed using Syto 13 (Molecular Probes, Eugene, Oregon, USA) and propidium iodide (PI) staining and a fluorescence-equipped microscope (Olympus, SELI, Toulouse, France) as previously described [21].

A mixture of freshly isolated healthy T lymphocytes and Jurkat T cells was treated by photochemotherapy. The percentage of each cell type was determined before and after PCT treatment by flow cytometry, using the cell size and CD1d mAb staining (BD Pharmingen, San Diego, CA, USA). T lymphocytes were defined as CD1d-negative cells with low size and Jurkat T cells as CD1d-positive cells with a greater size. In some experiments, 24 h after PCT the mixture was incubated for 48 h with anti-CD28 mAb (1 µg/mL; clone L283, Becton Dickinson), anti-CD3 mAb (10 ng/mL; Orthoclone, Janssen-Cilag, Issy-les-Moulineaux, France) and Interleukin-2 (100 UI/mL; BD Pharmingen). Then, T lymphocyte activation was checked by flow cytometry using a FITC-labelled anti-CD25 mAb (BD Pharmingen) in the CD3+/CD1d- lymphocyte population.

### Western blot analysis

Protein extracts (20 µg) were separated on SDS-PAGE and blotted on nitrocellulose membranes. The blots were analyzed using anti-cleaved PARP, anti-caspase 9 and anti-caspase 3, anti-LC3B (Cell Signaling Technology, Danvers, MA) or anti-ß-actin (Sigma) antibodies, as described elsewhere [22]. Proteins extracted from FasL-treated Jurkat T cells served as a control for caspase-dependent cell death [18] whereas those from Jurkat cells cultured for 24 h in HBSS medium (without amino acids and FCS, Gibco) were used as a control for starvation-induced autophagy [23]. To quantify the bands obtained by Western blot analysis of LC3B proteins, the ImageJ software (http://rsb.info.nih.gov/ij/) was used. The ratio between areas under the curve (AUC) for LC3-II and LC3-I was calculated for each condition.

### ROS and ceramide production

ROS (Radical Oxygen Species) production was assessed as previously described [24] using the H_2_DCFDA probe (Invitrogen, Cergy Pontoise, France). Ceramide levels were measured as previously reported [25], using recombinant DAG kinase (a kind gift from Drs. D Perry and YA Hannun; Charleston, NC, USA).

### Statistical analysis

Results are expressed as the means ± SD or SEM of data obtained from at least 3 independent experiments. Statistical significance, determined by means of Student's *t*-test and/or Anova test, was considered when P<0.05.

## Results

### The TrMPyP-MorG conjugate triggers O-glycan-dependent cell death of Tn-positive Jurkat cells after white-light irradiation

**Figure 1:**
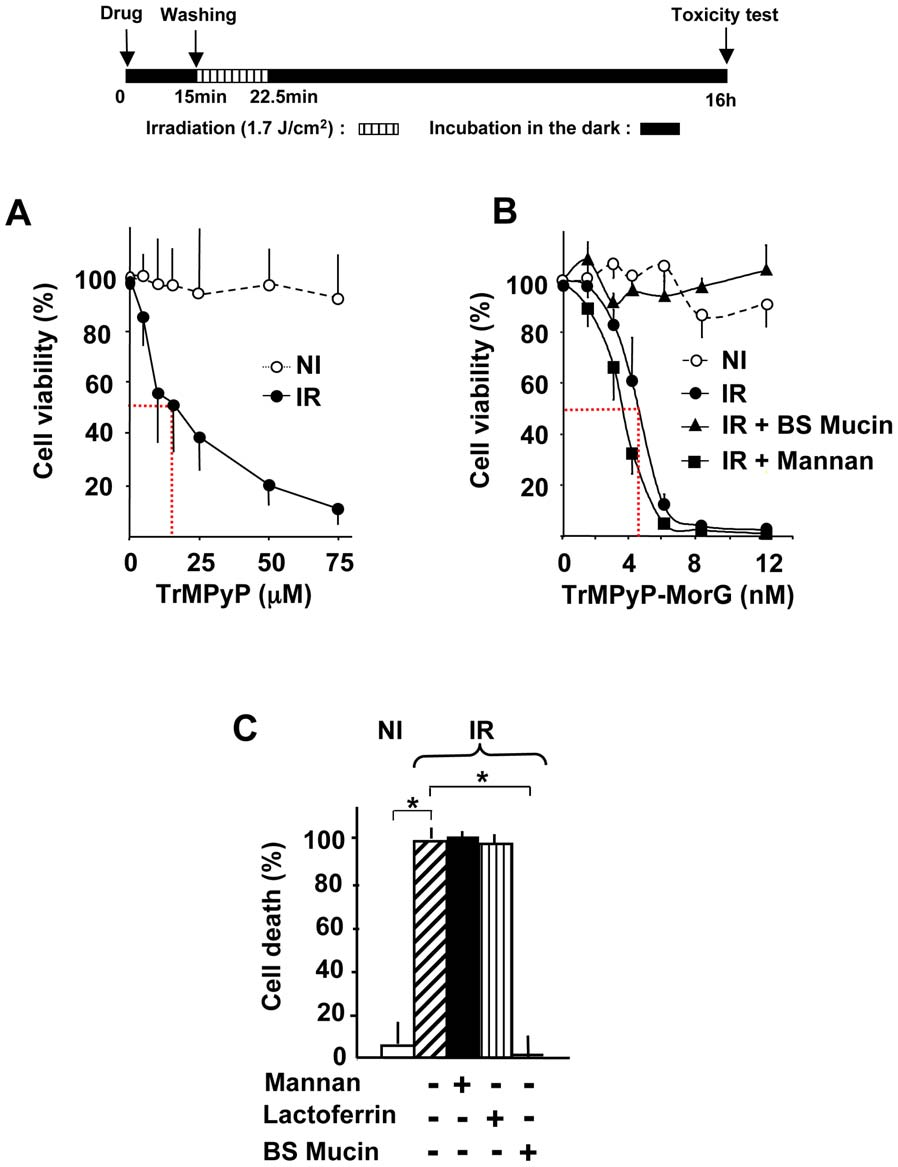
*In vitro* evaluation of O-glycan dependency of TrMPyP-MorG-mediated phototoxicity. Jurkat T cells (10^6^/mL) were incubated with various TrMPyP or TrMPyP-MorG concentrations for 15 min at 37°C in the dark. After washing, cells were irradiated (IR) or not (NI) by white light for 7.5 min (allowing a 1.7 J/cm^2^ light dose), and the cell viability was evaluated using MTT assay after 16 h incubation at 37°C in the dark. (**A**) Toxicity of free TrMPyP. (**B**) Cells were incubated with or without TrMPyP-MorG in the presence of Bovine Submaxillary Mucin (BSM) or Mannan (1 mg/mL). Of note, in the absence of irradiation no significant toxicity was observed at any TrMPyP-MorG concentrations (Anova test). (**C**) Protective effect of BSM (1 mg/mL) as compared to Lactoferrin (a N-glycosylated glycoprotein, 1 mg/mL) and Mannan on TrMPyP-MorG (15 nM)-induced PCT. Results are means ± SD from 3–5 independent experiments. * P<0.001, Anova test, as compared to IR (as well as to IR+ Mannan or IR+ lactoferrin).

Previously, using various concentrations of TrMPyP-MorG we demonstrated that a 10 nM concentration induced >90% phototoxicity on Jurkat leukemia cells, whereas 10 nM of free TrMPyP was non toxic [16]. To specify: *(i)* the dose effect of the conjugate after PCT and *(ii)* the dependency of O-glycosylation recognition for TrMPyP-MorG-mediated phototoxicity, cells were incubated for 15 min with various conjugate concentrations and with or without complex glycosylated molecules (Figure 1). Under our experimental conditions, with or without white-light irradiation, the free lectin seemed not toxic up to 1 µM (Figure S1). While no toxicity was noticed after incubation with free porphyrin or TrMPyP-MorG in the absence of irradiation (NI), a dose-dependent cell death was observed with both agents after irradiation (IR) (Figure 1A and B). Of special interest was the finding that, whereas a LD_50_ close to 15 µM was observed for free porphyrin (Figure 1A), a LD_50_ of 5 nM was found using the TrMPyP-MorG complex (Figure 1B), indicating a porphyrin phototoxicity increased by at least a factor of 1000. During incubation with the conjugate, the addition of Bovine Submaxillary Mucin (BSM) totally inhibited cell death, whereas addition of mannan (a mannose polymer, Figure 1B) or lactoferrin (an only N-glycosylated glycoprotein, Figure 1C) did not prevent Jurkat cell death. Thus, because BSM is a glycoprotein containing high density O-linked cryptic polyvalent T/Tn glycotopes, the phototoxicity induced in Jurkat cell line by the TrMPyP-MorG conjugate is probably dependent of O-glycosylation specific recognition and probably of T/Tn antigens at the cell surface.

### TrMPyP-MorG conjugate-mediated cytotoxicity allows selective depletion of Jurkat cells and killing of various leukemia cells

**Figure 2:**
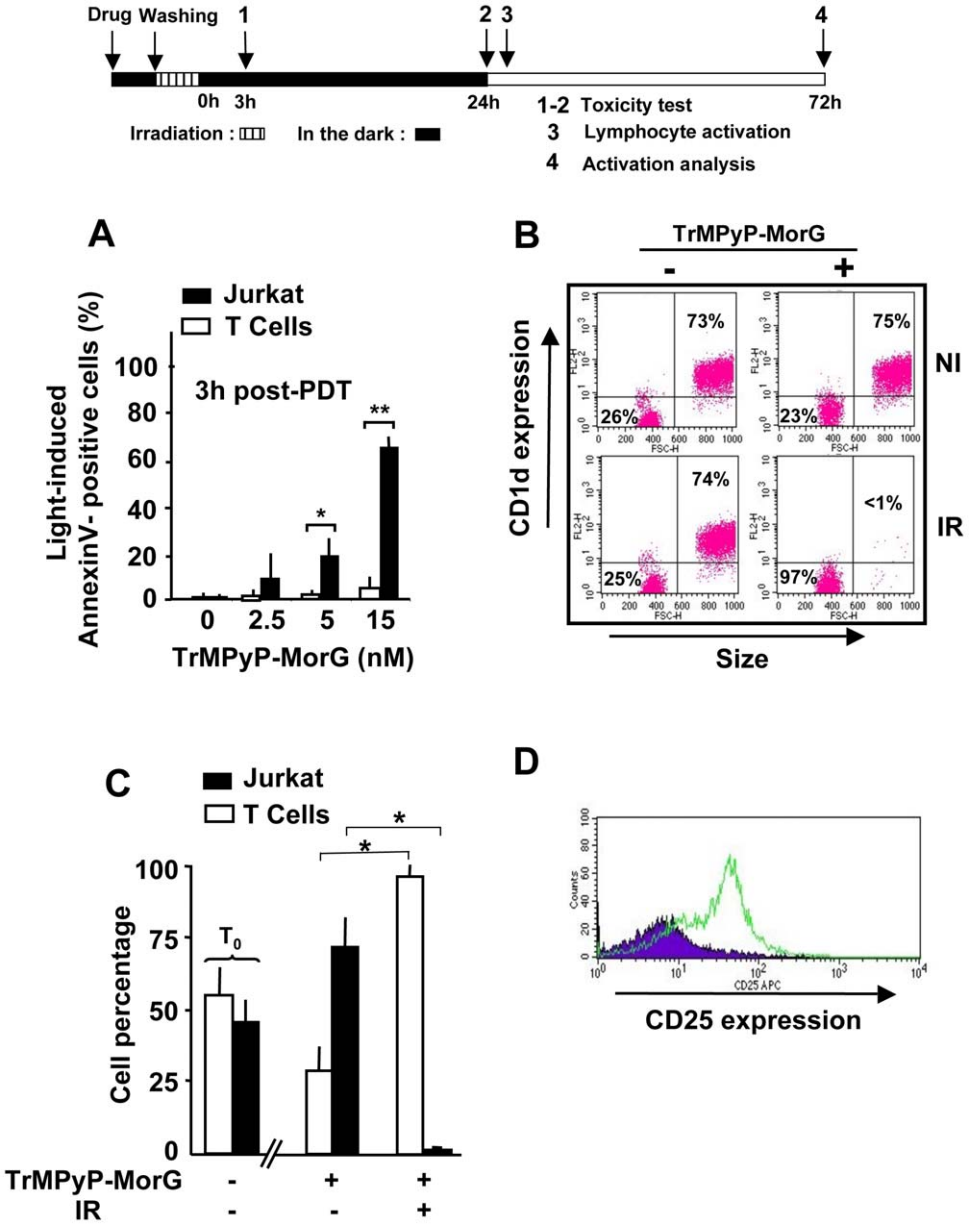
Effect of TrMPyP-MorG and PCT on a mixture of Jurkat leukemia T cells and healthy T lymphocytes. (**A**) T lymphocytes isolated from healthy donors and Jurkat T cells were incubated with TrMPyP-MorG and irradiated as described in the legend to Figure 1. Three hours after white light exposure (1.7 J/cm2), cells were stained by Annexin V-FITC and analyzed by flow cytometry. Results are means ± SD of 3 independent experiments. ** P<0.01 and * P<0.05 as calculated using student t test. Application of Anova test showed a very significant (P = 0.0026) difference in the susceptibility of Jurkat and T lymphocytes to TrMPyP-MorG-induced phototoxicity. (**B–D**) T lymphocytes (10^6^/mL) from healthy donors were mixed with Jurkat T cells (10^6^/mL), then treated or not by TrMPyP-MorG (15 nM) and white light (1.7 J/cm2). (**B–C**) The cell mixture was analyzed by flow cytometry after 24 h culture. As compared to T lymphocytes, Jurkat T cells were characterized by a higher size and by CD1d positivity. (**B**) Representative experiment, (**C**) means ± SD of results from 4 independent experiments. T_0_ shows the percentage of the two cell types in the cell mixture before TrMPyP-MorG addition. Significant changes (* P<0.001, Anova test) in cell percentage before and after irradiation were observed. (**D**) 24 h post-PCT, the TrMPyP-MorG treated cell mixture was incubated for 48 h with (empty histograms) or without (full histograms) IL-2+anti-CD3 and anti-CD28 monoclonal antibodies. Then CD3+/CD1d- lymphocytes were analyzed by flow cytometry for CD25 expression as a marker for lymphocyte activation.

The T lymphocyte-surface glycosylation is modified during T-cell development as well as T-cell activation [26] or oncogenesis [27]. Human Jurkat leukemia cells are known to have a T lymphocyte phenotype (for instance, they express a CD3 complex and a α/β TCR) but an aberrant glycosylation. Indeed, its O-glycosylation pattern is characterized by an overxepression of CD175 antigen, i.e. Tn antigen, due to UDP-Gal:GalNAc α1-ser/thr β1–3 galactosyl-transferase deficiency [28]. On the contrary, normal T lymphocytes are CD175-negative [7]. To further evaluate the targeting potential of the TrMPyP-MorG conjugate, its toxicity was comparatively tested against Jurkat leukemia T cells and isolated peripheral T lymphocytes. Previously, it was observed that healthy T lymphocytes slightly bound a fluorescein-labelled MorG whereas Jurkat leukemia T cells strongly bound it [16]. Of particular interest was the finding that Jurkat leukemia T cells were significantly more susceptible to PCT-induced cell death than normal T cells at 5 nM (the LD_50_) and 15 nM concentrations (Figure 2A), suggesting that the therapeutic index of the conjugate could be sufficient to preserve healthy lymphocytes from phototoxicity in the blood of leukemic patients, with the perspective of extracorporeal photochemotherapy. When PCT was applied to a 1∶1 mixture of Jurkat T cells and T lymphocytes, virtually all the leukemic cells were killed whereas healthy T cells survived, at least during the 24 h post-treatment (Figure 2 B and C). Of note, in the absence of irradiation, the percentage of normal T-lymphocytes in the mixture decreased to 25% after 24 h of culture, because Jurkat cells continued to grow while naïve lymphocytes did not (Figure 2C). Interestingly, after irradiation the healthy lymphocytes remained functionally competent, as CD25 expression increased 72 h after PCT upon immune stimulation (Figure 2D).

**Figure 3:**
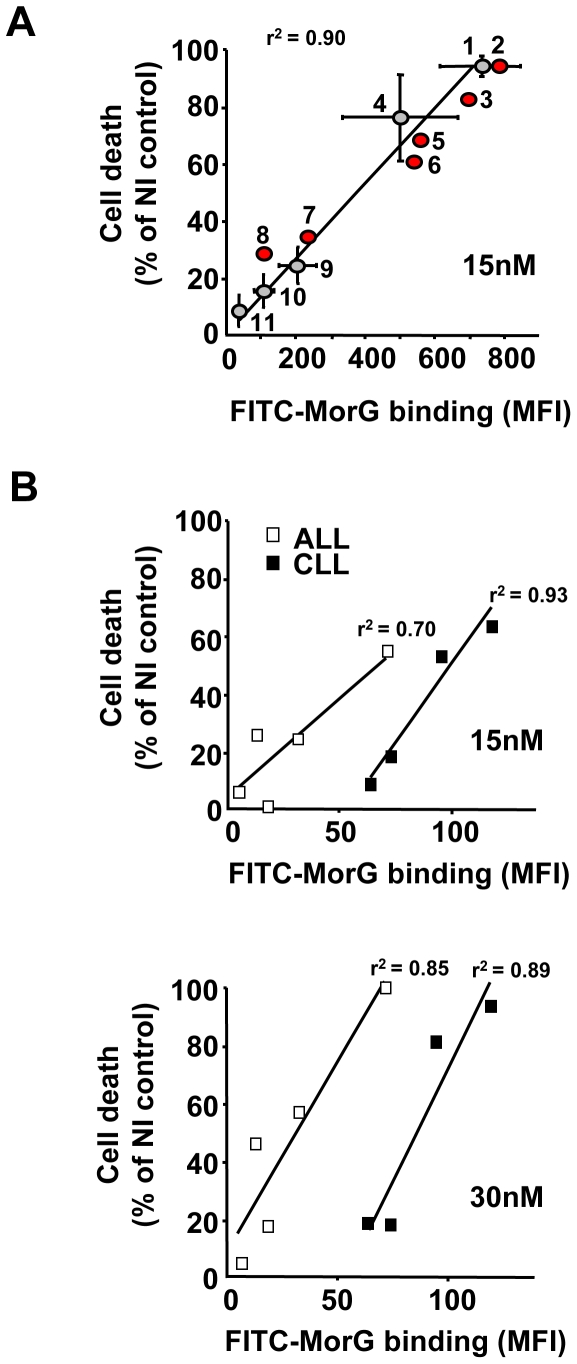
Effect of TrMPyP-MorG and PCT on human hematopoietic cell lines and leukemia cell samples. Human cell lines or fresh samples from patients with acute (ALL) or chronic (CLL) lymphoid leukemia were treated with TrMPyP-MorG and white light (1.7 J/cm^2^), as previously indicated for Jurkat T cells, and cell viability was determined using MTT assay 24 h after irradiation. Cell death is expressed as a percentage of non-irradiated control cells. Concomitantly, the binding (30 min at 4°C) to the cells of FITC-MorG (0.125 µg/mL) was evaluated by flow cytometry (Mean flurorescence intensity (MFI) was measured). (**A**) Correlation between lectin binding and phototoxicity after treatment with 15 nM TrMPyP-MorG using Jurkat (1), Molt 4 (3) and CEM (7) (T lymphoid leukemia cell lines), HuT78 (9) (a Sezary T lymphoma cell line), ERG (4) and SKW 6.4 (6) (EBV-transformed B lymphoid cell lines), KG1a (2), KG1 (5), HL60 (8), K562 (10), U937 (11) (myeloid leukemia cell lines) cells. (**B**) Correlation between lectin binding and phototoxic effect after treatment with 15 nM or 30 nM TrMPyP-MorG of fresh primary ALL and CLL from five and four different patients, respectively. Results are the means ± SEM of 3–6 independent experiments for cell lines 1, 4, 9, 10 and 11, means of 2 independent experiments for other cell lines, and the mean of quadruplicate determination for leukemia samples. r^2^ = linear correlation index.

Using hematopoietic cell lines of various origins, PDT-induced toxicity of TrMPyP-MorG was directly correlated with the level of FITC-labelled MorG binding [as evaluated at 4°C, as previously described [16]] to the cells (Figure 3A). Two different sets of cell lines could be distinguished. The first group with high MorG binding, which exhibit a high sensitivity to phototoxicity, included Jurkat and Molt4 cells (two T-lympoid leukemia cell lines), KG1 and KG1a (two myeloid leukemia cell lines) and ERG and SKW6.4 cells (two EBV-transformed B lymphoid cells). The second group showed little sensitivity or even resistance to phototoxicity.

Because PCT is currently used in the clinic to treat T cell lymphoma in ECP protocols, the conjugate was evaluated against various lymphoid malignancies. The incubation of different acute (ALL) or chronic (CLL) lymphoid leukemia primary cells from patients confirmed that PCT-induced toxicity (as assessed by MTT) increased with the TrMPyP-MorG dose and was correlated with the level of MorG-binding on tumor cells, supporting the notion that treatment efficiency mainly depends on lectin-binding at the cell surface (Figure 3B). When the number of patients' primary leukemic cells was large enough, the toxic effect was further confirmed using annexin V-FITC/PI staining (Figure S2).

### The TrMPyP-MorG conjugate kills Jurkat T leukemia cells mainly through a caspase-independent pathway

**Figure 4:**
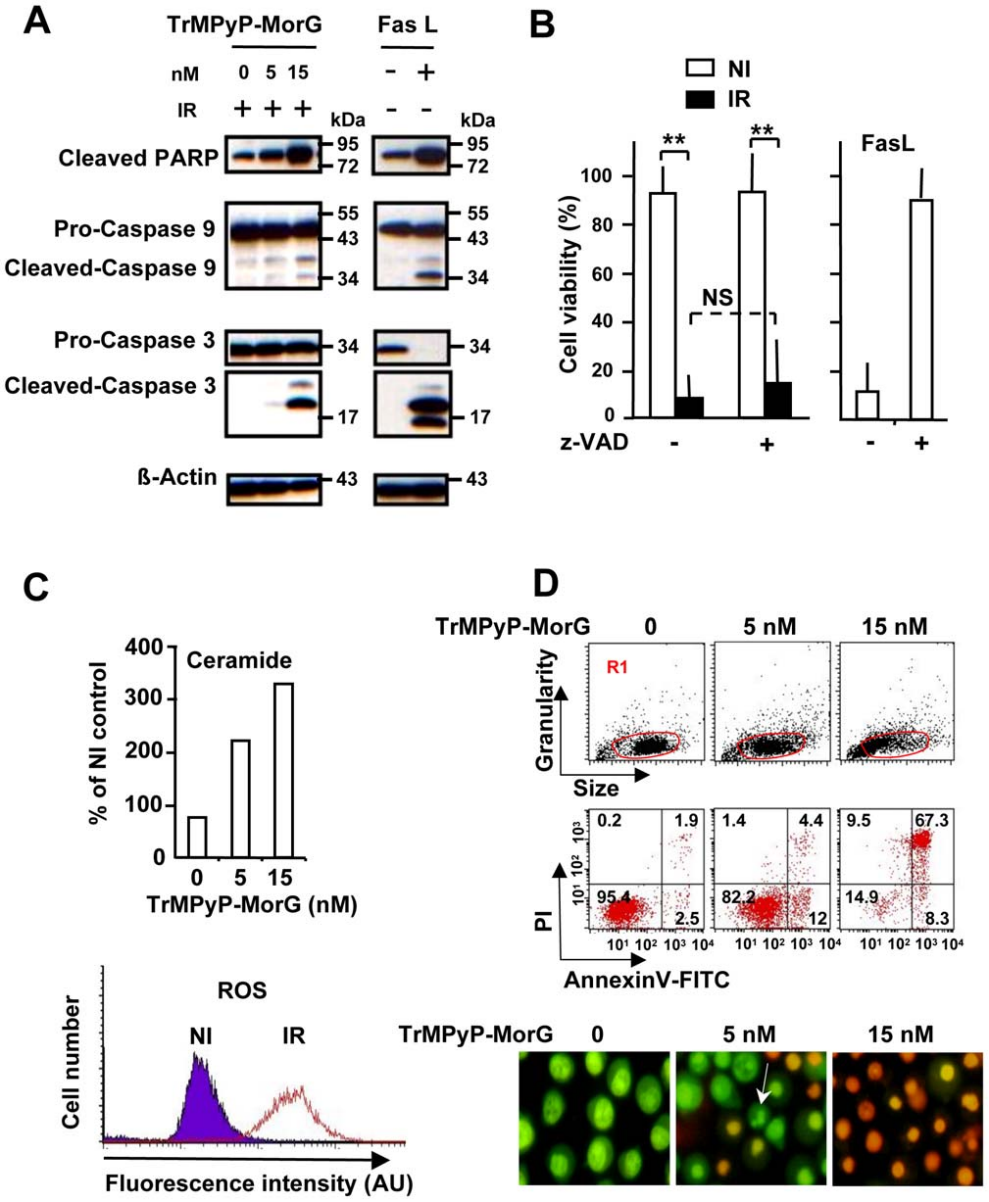
Cell death signaling triggered by PCT using TrMPyP-MorG conjugate. Jurkat T cells (10^6^/mL) were incubated with various TrMPyP-MorG concentrations for 15 min at 37°C and in the dark. After washing, cells were irradiated (IR) or not (NI), for 7.5 min. (**A**) Western blot analysis of cells 3 h post-irradiation. FasL (50 ng/mL) was used as a control for inducing apoptosis. (**B**) Jurkat T cells were incubated in the presence (+) or absence (−) of 20 µM of the pan-caspase inhibitor (z-VAD), then treated by TrMPyP-MorG (15 nM) and irradiated (IR) or not (NI). FasL-induced cell death was used as a control. Cell death was analyzed using MTT assay, 24 h after PCT. Results are means ± SD of 4 separate experiments (similar results were obtained with 5 nM TrMPyP-MorG), ** P<0.001, Anova test; NS, not significant. (**C**) Intracellular ceramide concentration was determined 3 h after PCT. Results are expressed as percentage of the ceramide levels in drug-treated controls without irradiation (mean of two separate experiments; similar results were observed 24 h post-PCT). Cellular ROS levels were analyzed using flow cytometry. Representative results after TrMPyP-MorG (5 nM) treatment and 15 min post-irradiation. (**D**) Three hours after irradiation, cells were stained with AnnexinV-FITC/PI (upper panel) or Syto13/PI (lower panel) and cell death was evaluated by flow cytometry (in gate R1, defined to exclude cell debris from the analysis) or by fluorescence microscopy, respectively. The arrow indicates a cell with classical features of apoptosis (reduction of cellular volume, nuclear fragmentation, PI exclusion).

**Figure 5:**
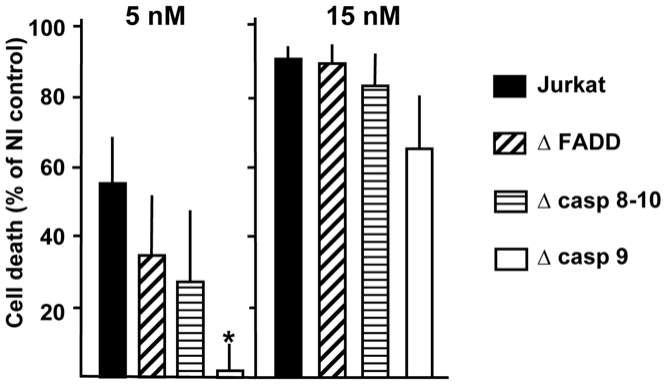
Defects in the caspase signalling pathway do not protect from TrMPyP-MorG conjugate phototoxicity. Parental Jurkat T cells (Jurkat), FADD-deficient (Δ FADD), caspase 8- and 10-doubly deficient (Δ casp 8–10) or caspase 9-deficient (Δ casp 9) Jurkat cells (10^6^/mL) were incubated with TrMPyP-MorG (5 or 15 nM) for 15 min at 37°C in the dark. After washing, cells were irradiated by white light (7.5 min, 1.7 J/cm2) and the cell viability was evaluated using MTT assay after 16 h incubation at 37°C in the dark. Results are means ± SEM from 3–6 independent experiments, P*<0.05 as compared to parental Jurkat T cells.

**Figure 6:**
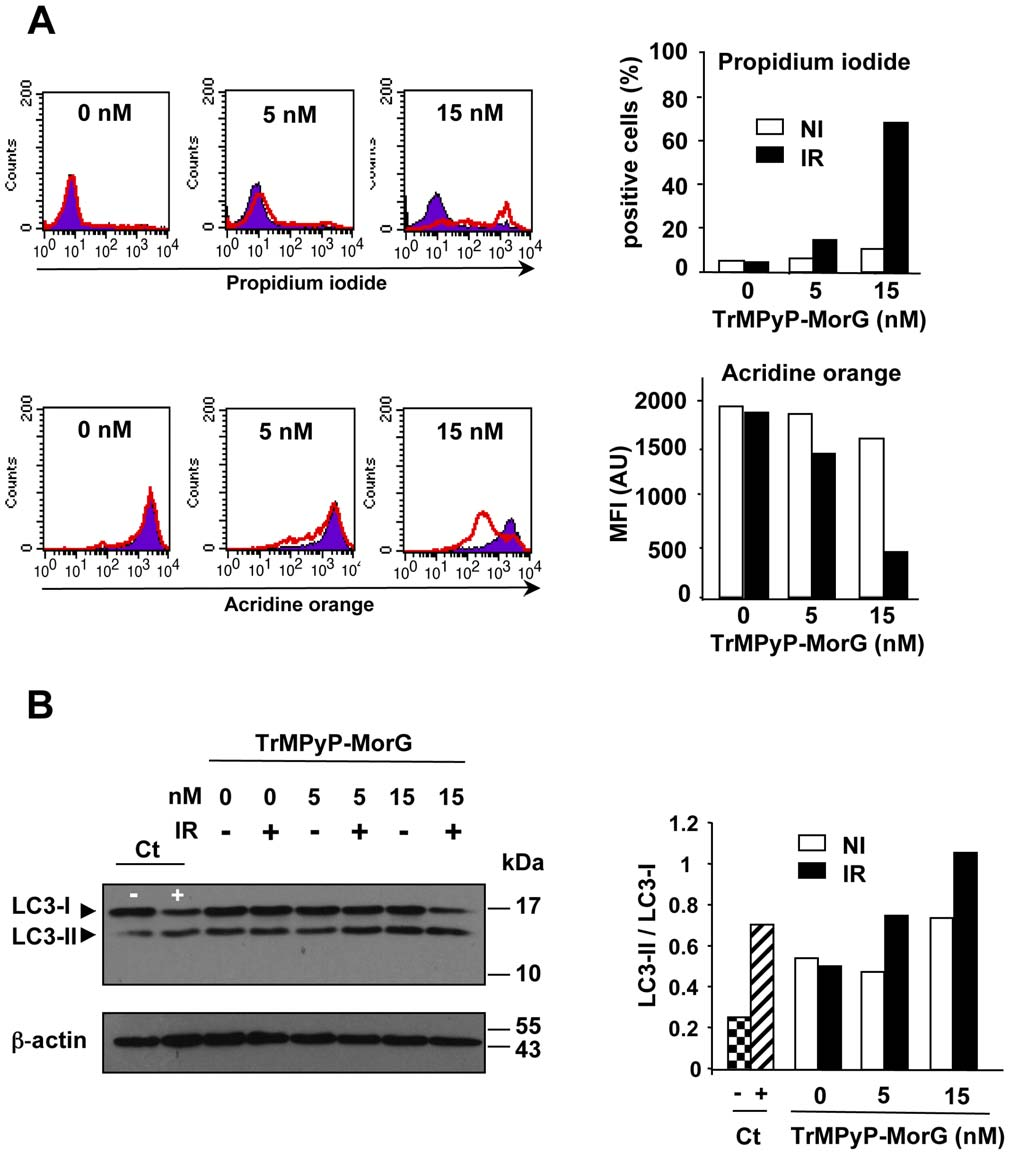
Induction of necrosis by TrMPyP-MorG conjugate. Parental Jurkat T cells (10^6^/mL) were incubated with TrMPyP-MorG (5 or 15 nM) for 15 min at 37°C in the dark. After washing, cells were irradiated (IR) or not (NI) by white light (7.5 min, 1.7 J/cm2) and incubated 3–4 h at 37°C in the dark. (**A**) The cell viability was evaluated using PI staining, whereas the formation of acidic vesicular organelles (AVO) was assessed by using acridine orange. Cells were analyzed by flow cytometry excluding cell debris. Left, representative histograms: NI cells, full histograms; IR cells, empty histograms. Right, means of two independent experiments. (**B**) Left, representative Western blot to detect the conversion of LC3-I to LC3-II. Right, densitometry analysis; results are means of two independent experiments. Control cells (**Ct**) were cultured for 2 days either in RPMI medium containing 10% FCS (**−**) or HBSS medium (without amino acids and FCS) (**+**), a condition known to induce autophagy and AVO formation.

To analyse the mechanisms of cell death triggered by the conjugate, experiments were conducted to test whether an apoptotic, caspase-dependent pathway was activated. As a control for apoptosis, Jurkat cells were treated with FasL (and without irradiation). Western blot analysis demonstrated the cleavage of caspase 3 (effector caspase) and caspase 9 (initiator caspase) and PARP (a nuclear target of effector caspase), indicating that TrMPyP-MorG used in PCT triggered caspase activation (Figure 4A). However, when a pan caspase-inhibitor (zVAD-fmk) was added, no significant inhibition of cell death was observed, suggesting the limited involvement of caspases in the phototoxic effect of the conjugate (Figure 4B). Accordingly, variants of Jurkat cells being deficient for different proteins of the caspase signalling pathway [i.e. FADD (Fas-Associated protein with Death Domain)-, caspase 9-, as well as caspase 8 and 10 doubly-deficient cells] were not significantly protected from TrMPyP-MorG-induced cell death, even though caspase 9-deficient Jurkat cells were resistant to 5 nM, but not 15 nM, of conjugate (Figure 5). In addition, TrMPyP-MorG conjugate-induced cell death was characterized by an increase of both intracellular ROS and ceramide levels (Figure 4C). Finally, TrMPyP-MorG induced cell death was associated with: *(i)* phosphatidylserine externalization (*i.e.*, AnnexinV-FITC staining) (Figure 4D, high), (*ii*) membrane permeability increase (*i.e.*, PI staining), and (*iii*) a combination of morphological features of necrosis (PI staining) and apoptosis (chromatin condensation, nuclear fragmentation) (Figure 4D, bottom). When TrMPyP-MorG-treated cells were stained with PI or acridine orange (AO), the irradiation resulted in a strong PI staining together with a decrease of AO fluorescence (Figure 6A). In normal cells, when retained in lysosomes AO traps the protons and emits a red fluorescence. Induction of autophagy is characterized by AVO formation and an increased AO red fluorescence emission [20], [23]. Our present findings suggest that the increase of plasma membrane permeability is accompanied by a loss of lysosomal membrane integrity and the release of the accumulated AO from lysosomes. Nonetheless, under our experimental conditions, irradiation of conjugate-treated cells induced a modest increase of LC3-II level, known to be an autophagic marker (Figure 6B).

## Discussion

The present data demonstrate that plant lectins could serve as photosensitizer carrier proteins for the targeting of aberrant glycosylation on tumor cells and, with regard to antibodies, as an interesting additional weapon. Indeed, monoclonal antibodies are the most successful binding molecules in biomedicine, but their use is still limited in drug targeting, for instance by the difficulty and expensiveness of manufacturing, and the relative instability under conditions of covalent coupling with drug [29]. Conversely, the structure of plant lectins confers strong resistance to heat and chemical denaturing [12], [13]. For instance, MorG presents a good thermodynamic stability, partially due to the absence of cysteines and consequently of disulfide bonds [30]. The extraction of the lectin from the bark of black mulberry tree is relatively simple and the chemical linkage with PS is easy. In addition, this lectin, as others, could be probably prepared by bioengineering [31]. Finally, such a coupling with plant lectin appears to be commercially realistic. With the aim to develop PS-targeting strategy, different immunoconjugates have previously been prepared between monoclonal antibodies [*i.e.*, against colorectal antigens [32], [33] or HER2 antigen [34], [35]] and different PS molecules. Obviously, PS immunoconjugates are more selective for target cells than free PS, but can be less phototoxic on a per mole basis [34], [36]. On the contrary, the TrMPyP-MorG conjugate used here strongly increased the PS phototoxicity, for instance at least of 1000 times on Jurkat leukemia cells. In addition, to our knowledge no conjugate has been prepared between a PS and a monoclonal antibody against T/Tn antigens. Yet, several anti-T and anti-Tn antibodies have been generated but with inconsistent results in their anti-tumor activities. A possible explanation is that previously prepared monoclonal antibodies recognize a conformational epitope constituted both by a peptide and an altered glycan, rather than the sole oligosaccharide moiety [10]. Consequently, these monoclonal antibodies can, at least partly, cross react with the target peptide on normal cells having a normal glycosylation. In contrast, some plant lectins, such as MorG have a high affinity for specific carbohydrate structure, independently of the peptide that carries the carbohydrate moiety structures. As altered glycans are over-expressed on tumor cells, plant lectins represent a powerful tool to discriminate between normal and cancer cells, by the mean of their cell surface binding to certain glycotopes, *e.g.* clusters of T/Tn antigens. Hence, our present data suggest that a plant lectin could be used as an efficient carrier for PS targeting, specifically into tumor cells expressing alterations of glycosylation, actually O-glycosylation. As other lectins, MorG recognizes different sets of glycotopes (high affinity to T and Tn structures), but can discriminate between subtle glycosylation modifications [14], [15], for instance in the present work, alterations of cell surface glycoconjugates between leukemic and healthy T lymphocytes.

Our observations demonstrate for the first time that coupling a porphyrin with a plant lectin allows the selective elimination of leukemic cells mixed with healthy T cells, while fully preserving the functionality of normal T cells. Using different hematopoietic cell lines as well as primary lymphoid leukemia cells, the extent of cell death induced by treatment correlated with the level of MorG binding on target cells. Consequently, a useful tool to estimate the tumor sensitivity to conjugate-mediated cytotoxicity might be the determination of the level of lectin binding at 4°C on the target cell surface. However, in spite of a FITC-MorG binding lower than in sensitive leukemic cell lines, some fresh lymphoid leukemia cells were sensitive to the conjugate-mediated phototoxicity, quite similarly to the sensitive established cell lines (Figure 3B). In addition, because cell lines with T lymphoid, B lymphoid or myeloid phenotype were similarly killed (Figure 3A), the present data suggest that the susceptibility of leukemia cells to conjugate-mediated toxicity is independent of the lymphoid or myeloid phenotypes. The degree of sensitivity of tumor cells might be related to the biochemical intracellular characteristics (such as intrinsic resistance mechanisms to cell death) rather than to the hematopoietic phenotype of leukemia cells. The results obtained with fresh leukemia samples suggest that, as compared to ALL cells, a higher binding of the lectin is required for killing CLL cells by the MorG conjugate (Figure 3 and Figure S2). Because the increase of ROS concentration is an essential step for PCT-induced cell death using porphyrins [37], one possible explanation might be that the status of oxidative stress and/or antioxidant enzymes of ALL and CLL cells is different. ALL samples are characterized by a decrease of anti-oxidants (for instance, a decrease of catalase and superoxide dismutase activities [38], [39]) and a sustained oxidative stress (due to a low NAD(P)H:quinone oxidoreductase 1 activity, known to attenuate oxidative stress [40], and/or aggressive chemotherapy protocols [38]). CLL are long-lived cells *in vivo*, developing clonal resistance to apoptosis through distinct mechanisms, such as overexpression of anti-apoptotic Bcl-2 family members [41], [42]. CLL cells also appear to have a complex susceptibility to oxidative stress, being able to express and release catalase extracellularly [43].

Regarding the intracellular signaling mechanisms of TrMPyP-MorG-induced toxicity after light irradiation, a previous work indicates that cells quickly (5 min) took up the conjugate [16]. Whether only the internalized conjugate or, additionally, the cell surface bound conjugate contributes to cytotoxicity needs to be investigated. The present results suggest that caspase activation was not absolutely required for efficient cell death after light irradiation, as previously shown for Foscan®-mediated phototoxicity [44]. It is possible that the conjugate triggers, in the same cell, both caspase-independent and caspase-dependent events. Thus, the PARP-1 dependent cell death program can be triggered by ROS, independently of caspases [45], [46]. In agreement with other previous observations concerning the mechanisms of cytotoxicity mediated by photoactivation of porphyrins or similar PS, treated target cells exhibited an increase in ceramide and ROS concentrations, two putative second messengers of cell death [47], [48], which can be involved in caspase-dependent and caspase-independent cytotoxic processes [49]. The ROS are proposed as the main agents triggering the demise process of tumor cells after PDT [49]. They can kill tumor cells directly by apoptosis and/or necrosis [50]. In the present work, the TrMPyP-MorG-mediated phototoxicity was characterized by several necrosis features, such as caspase-independence and plasma membrane permeability. In addition, the decrease of AO staining induced by TrMPyP-MorG treatment might be due to lysosomal disruption, in agreement with recent data indicating that necrotic cell death can be characterized by irreversible lysosomal permeabilization [51]. The elevated level of LC3-II we observed might be compatible with a necrotic process since necrosis-initiating insults can result in activation of autophagy [52]. Thus, a main mechanism for TrMPyP-MorG-mediated phototoxicity could be a necrotic cell death, at least in Jurkat T cells. It is tempting to speculate that irradiation of TrMPyP-MorG- treated cells might induce a strong oxidative stress that triggers lysosomal membrane rupture and subsequent necrosis, as proposed for neuronal death [52]. Finally, taken together, our data suggest that PCT-induced toxicity using TrMPyP-MorG can bypass the caspase pathway to trigger cell death. This might be a valuable advantage over apoptosis-inducing agents, since tumor cells often resist caspase-dependent cell death [53].

ECP is currently used for treating T lymphoma (Sezary lymphoma). This PCT method uses UV-A radiation and non-targeted PS (psoralen) but does not preserve the healthy leukocytes that are mixed with the malignant lymphocytes. The efficiency and selectivity of PCT we observed using TrMPyP-MorG on a mixture of leukemic and healthy cells suggest that this type of conjugate could be advantageously employed in ECP against some chronic hematopoietic malignancies. Indeed, the use of such white-light activatable PS can allow lymphocyte preservation because: *i*) the PS is targeted to cells exhibiting abnormal glycosylation; *ii*) white-light is surely less toxic than UV on healthy lymphocytes. Consequently, the new approach we describe could be used in order to reduce the tumor mass and protect healthy lymphocytes, preserving the ability of anti-tumor immune response. Indeed, re-infusion of PCT-treated leukemic cells might trigger auto-vaccination mechanisms [1], [3], [5]. In addition, the level of Tn antigen expression and of MorG binding was significantly higher on activated T lymphocytes than on resting T lymphocytes (Figure S3), suggesting that PS-MorG conjugates might be used to eliminate abnormal activated-lymphocytes by ECP. Indeed, increasing amounts of observations indicate that ECP can be useful to treat patients with autoimmune diseases or with chronic graft versus host disease, which are all characterized by the presence of abnormal activated-T cells [54].

In conclusion, whereas further *in vivo* experiments need to be performed to document the efficiency of the PS-lectin conjugate, the present observations demonstrate the potential of plant lectins to target glycan alterations on tumor cells and to enhance the delivery of photosensitizers in PCT *in vitro*, *e.g.* in ECP and potentially in PDT. Plant lectins might also be used to increase PDT efficiency against solid tumors, such as T/Tn positive epithelial tumor cells. In addition, some plant lectins have specific affinity for binding to the inflamed or non-inflamed endothelial cells [55]. As targeting in PDT is mainly achieved today through precise application of the light, conjugation of PS with a lectin able to target tumor vasculature could enhance the efficiency of this treatment in oncology.

## Supporting Information

Figure S1 **Toxicity of free MorG.** Jurkat T cells (10^6^/mL) were exposed to various concentrations of MorG (from 5 to 5000 nM) for 15 min at 37°C, then washed. Cells were irradiated (**IR**) or not (**NI**) as indicated in material and methods. Cell viability was evaluated using MTT assay after 24 h of culture. Results are means ± SD of 3 independent experiments. The rectangle corresponds to range of concentrations used for the evaluation of conjugate phototoxicity in the present work. * P<0.05, as compared to 5 or 100 nM TrMPyP-MorG.
(TIF)

Figure S2 **The toxic effect of conjugate on fresh primary leukemia cells was confirmed using annexin V-FITC/PI staining and flow cytometry analysis.** Fresh samples from patients with acute (**ALL**) or chronic (**CLL**) lymphoid leukemia were treated with TrMPyP-MorG and white light (1.7 J/cm^2^), as indicated in Figure 3. When the sample size permitted, the cell viability was determined 3–4 hours after irradiation using AnnexinV-FITC/PI staining and analysis by flow cytometry (in gate R1, defined to exclude cell debris from the analysis or in gate R2 defined as containing apparently viable cells, i.e. corresponding to normal morphology of lymphocytes and lymphoblastes, in size and granularity). (**A** and **B**) Representative experiment analysis of a CLL sample : (**A**) in gate R1 and (**B**) in gate R2. Some fresh samples contained relatively high proportion of dead cells, but allowed evaluation of conjugate-induced cell death from the percentage determination of viable cells (defined as AnnexinV-FITC and PI negative cells). (**C** and **D**) Correlation between lectin binding and phototoxic effect after treatment with 15 nM or 30 nM TrMPyP-MorG of fresh primary ALL and CLL from four and three different patients, respectively. Results are the means of duplicate determination for each leukemia samples in gate R2.
(TIF)

Figure S3 **Comparative binding of MorG on resting T lymphocytes and activated-T lymphocytes.** T lymphocytes isolated from healthy donors were incubated for 48 h with anti-CD3 and anti-CD28 monoclonal antibodies and lymphocyte activation was controlled using staining with anti-CD25 monoclonal antibody. Activated T cells, resting T cells from the same donor and Jurkat T cells were incubated with FITC-MorG (0.25 µg/mL) or anti-CD175 (anti-Tn) monoclonal antibody. (**A**) Representative experiment, autofluorescence: full histograms, positive fluorescence : empty histograms. (**B**) Mean fluorescence intensity (MFI) was measured. Results for MorG staining are shown, means of three experiments, Auto = autofluorescence, *P<0.05 between resting and activated T cells.
(TIF)

## References

[1] Oliven A, Shechter Y (2001). Extracorporeal photopheresis: a review.. Blood Rev.

[2] Dolmans DE, Fukumura D, Jain RK (2003). Photodynamic therapy for cancer.. Nat Rev Cancer.

[3] Kabingu E, Oseroff AR, Wilding GE, Gollnick SO (2009). Enhanced systemic immune reactivity to a Basal cell carcinoma associated antigen following photodynamic therapy.. Clin Cancer Res.

[4] Castano AP, Mroz P, Hamblin MR (2006). Photodynamic therapy and anti-tumour immunity.. Nat Rev Cancer.

[5] Korbelik M, Stott B, Sun J (2007). Photodynamic therapy-generated vaccines: relevance of tumour cell death expression.. Br J Cancer.

[6] del Carmen MG, Rizvi I, Chang Y, Moor AC, Oliva E (2005). Synergism of epidermal growth factor receptor-targeted immunotherapy with photodynamic treatment of ovarian cancer in vivo.. J Natl Cancer Inst.

[7] Cao Y, Merling A, Karsten U, Goletz S, Punzel M (2008). Expression of CD175 (Tn), CD175s (sialosyl-Tn) and CD176 (Thomsen-Friedenreich antigen) on malignant human hematopoietic cells.. Int J Cancer.

[8] Itzkowitz SH, Yuan M, Montgomery CK, Kjeldsen T, Takahashi HK (1989). Expression of Tn, sialosyl-Tn, and T antigens in human colon cancer.. Cancer Res.

[9] Heimburg-Molinaro J, Almogren A, Morey S, Glinskii OV, Roy R (2009). Development, characterization, and immunotherapeutic use of peptide mimics of the Thomsen-Friedenreich carbohydrate antigen.. Neoplasia.

[10] Brooks CL, Schietinger A, Borisova SN, Kufer P, Okon M (2010). Antibody recognition of a unique tumor-specific glycopeptide antigen.. Proc Natl Acad Sci U S A.

[11] Shan L (2004–2009). [Biotinylated anti-Tn MLS128 monoclonal antibody-125I-streptavidin].. Molecular Imaging and Contrast Agent Database (MICAD) [Internet].

[12] Liu B, Bian HJ, Bao JK (2010). Plant lectins: potential antineoplastic drugs from bench to clinic.. Cancer Lett.

[13] Wu AM, Lisowska E, Duk M, Yang Z (2009). Lectins as tools in glycoconjugate research.. Glycoconj J.

[14] Benoist H, Culerrier R, Poiroux G, Segui B, Jauneau A (2009). Two structurally identical mannose-specific jacalin-related lectins display different effects on human T lymphocyte activation and cell death.. J Leukoc Biol.

[15] Singh T, Wu JH, Peumans WJ, Rouge P, Van Damme EJ (2007). Recognition profile of Morus nigra agglutinin (Morniga G) expressed by monomeric ligands, simple clusters and mammalian polyvalent glycotopes.. Mol Immunol.

[16] Poiroux G, Pitié M, Culerrier R, Ségui B, Van Damme EJ (2010). Morniga G: a plant lectin as an endocytic ligand for photosensitizer molecule targeting towards tumor-associated T/Tn antigens.. Photochem Photobiol In Publication.

[17] Nyman ES, Hynninen PH (2004). Research advances in the use of tetrapyrrolic photosensitizers for photodynamic therapy.. J Photochem Photobiol B.

[18] Milhas D, Cuvillier O, Therville N, Clave P, Thomsen M (2005). Caspase-10 triggers Bid cleavage and caspase cascade activation in FasL-induced apoptosis.. J Biol Chem.

[19] Casas C, Saint-Jalmes B, Loup C, Lacey C, Meunier B (1993). Synthesis of cationic metalloporpyrin precursors related to the design of DNA cleavers.. J Org Chem.

[20] Chen Y, Azad MB, Gibson SB (2009). Superoxide is the major reactive oxygen species regulating autophagy.. Cell Death Differ.

[21] Alcouffe J, Therville N, Segui B, Nazzal D, Blaes N (2004). Expression of membrane-bound and soluble FasL in Fas- and FADD-dependent T lymphocyte apoptosis induced by mildly oxidized LDL.. Faseb J.

[22] Lafont E, Milhas D, Carpentier S, Garcia V, Jin ZX (2009). Caspase-mediated inhibition of sphingomyelin synthesis is involved in FasL-triggered cell death.. Cell Death Differ.

[23] Chen Y, Azad MB, Gibson SB (2010). Methods for detecting autophagy and determining autophagy-induced cell death.. Can J Physiol Pharmacol.

[24] Negre-Salvayre A, Auge N, Duval C, Robbesyn F, Thiers JC (2002). Detection of intracellular reactive oxygen species in cultured cells using fluorescent probes.. Methods Enzymol.

[25] Van Veldhoven PP, Matthews TJ, Bolognesi DP, Bell RM (1992). Changes in bioactive lipids, alkylacylglycerol and ceramide, occur in HIV-infected cells.. Biochem Biophys Res Commun.

[26] Toscano MA, Bianco GA, Ilarregui JM, Croci DO, Correale J (2007). Differential glycosylation of TH1, TH2 and TH-17 effector cells selectively regulates susceptibility to cell death.. Nat Immunol.

[27] Ohtsubo K, Marth JD (2006). Glycosylation in cellular mechanisms of health and disease.. Cell.

[28] Piller V, Piller F, Fukuda M (1990). Biosynthesis of truncated O-glycans in the T cell line Jurkat. Localization of O-glycan initiation.. J Biol Chem.

[29] Binz HK, Amstutz P, Pluckthun A (2005). Engineering novel binding proteins from nonimmunoglobulin domains.. Nat Biotechnol.

[30] Van Damme EJ, Hause B, Hu J, Barre A, Rouge P (2002). Two distinct jacalin-related lectins with a different specificity and subcellular location are major vegetative storage proteins in the bark of the black mulberry tree.. Plant Physiol.

[31] Lam SK, Ng TB (2010). Lectins: production and practical applications.. Appl Microbiol Biotechnol.

[32] Del Governatore M, Hamblin MR, Piccinini EE, Ugolini G, Hasan T (2000). Targeted photodestruction of human colon cancer cells using charged 17.1A chlorin e6 immunoconjugates.. Br J Cancer.

[33] Hamblin MR, Del Governatore M, Rizvi I, Hasan T (2000). Biodistribution of charged 17.1A photoimmunoconjugates in a murine model of hepatic metastasis of colorectal cancer.. Br J Cancer.

[34] Savellano MD, Pogue BW, Hoopes PJ, Vitetta ES, Paulsen KD (2005). Multiepitope HER2 targeting enhances photoimmunotherapy of HER2-overexpressing cancer cells with pyropheophorbide-a immunoconjugates.. Cancer Res.

[35] Serebrovskaya EO, Edelweiss EF, Stremovskiy OA, Lukyanov KA, Chudakov DM (2009). Targeting cancer cells by using an antireceptor antibody-photosensitizer fusion protein.. Proc Natl Acad Sci U S A.

[36] Savellano MD, Hasan T (2005). Photochemical targeting of epidermal growth factor receptor: a mechanistic study.. Clin Cancer Res.

[37] Price M, Reiners JJ, Santiago AM, Kessel D (2009). Monitoring singlet oxygen and hydroxyl radical formation with fluorescent probes during photodynamic therapy.. Photochem Photobiol.

[38] Mazor D, Abucoider A, Meyerstein N, Kapelushnik J (2008). Antioxidant status in pediatric acute lymphocytic leukemia (ALL) and solid tumors: the impact of oxidative stress.. Pediatr Blood Cancer.

[39] Battisti V, Maders LD, Bagatini MD, Santos KF, Spanevello RM (2008). Measurement of oxidative stress and antioxidant status in acute lymphoblastic leukemia patients.. Clin Biochem.

[40] Smith MT, Wang Y, Kane E, Rollinson S, Wiemels JL (2001). Low NAD(P)H:quinone oxidoreductase 1 activity is associated with increased risk of acute leukemia in adults.. Blood.

[41] Shanafelt TD, Lee YK, Bone ND, Strege AK, Narayanan VL (2005). Adaphostin-induced apoptosis in CLL B cells is associated with induction of oxidative stress and exhibits synergy with fludarabine.. Blood.

[42] Samuel S, Tumilasci VF, Oliere S, Nguyen TL, Shamy A VSV oncolysis in combination with the BCL-2 inhibitor obatoclax overcomes apoptosis resistance in chronic lymphocytic leukemia.. Mol Ther.

[43] Moran EC, Kamiguti AS, Cawley JC, Pettitt AR (2002). Cytoprotective antioxidant activity of serum albumin and autocrine catalase in chronic lymphocytic leukaemia.. Br J Haematol.

[44] Kessel D, Luo Y (1999). Photodynamic therapy: a mitochondrial inducer of apoptosis.. Cell Death Differ.

[45] Hong SJ, Dawson TM, Dawson VL (2004). Nuclear and mitochondrial conversations in cell death: PARP-1 and AIF signaling.. Trends Pharmacol Sci.

[46] Vittar NB, Awruch J, Azizuddin K, Rivarola V (2010). Caspase-independent apoptosis, in human MCF-7c3 breast cancer cells, following photodynamic therapy, with a novel water-soluble phthalocyanine.. Int J Biochem Cell Biol.

[47] Copley L, van der Watt P, Wirtz KW, Parker MI, Leaner VD (2008). Photolon, a chlorin e6 derivative, triggers ROS production and light-dependent cell death via necrosis.. Int J Biochem Cell Biol.

[48] Separovic D, Bielawski J, Pierce JS, Merchant S, Tarca AL (2009). Increased tumour dihydroceramide production after Photofrin-PDT alone and improved tumour response after the combination with the ceramide analogue LCL29. Evidence from mouse squamous cell carcinomas.. Br J Cancer.

[49] Buytaert E, Dewaele M, Agostinis P (2007). Molecular effectors of multiple cell death pathways initiated by photodynamic therapy.. Biochim Biophys Acta.

[50] Marchal S, Fadloun A, Maugain E, D'Hallewin MA, Guillemin F (2005). Necrotic and apoptotic features of cell death in response to Foscan photosensitization of HT29 monolayer and multicell spheroids.. Biochem Pharmacol.

[51] Giusti C, Luciani MF, Klein G, Aubry L, Tresse E (2009). Necrotic cell death: From reversible mitochondrial uncoupling to irreversible lysosomal permeabilization.. Exp Cell Res.

[52] Yamashima T, Oikawa S (2009). The role of lysosomal rupture in neuronal death.. Prog Neurobiol.

[53] Fadeel B, Ottosson A, Pervaiz S (2008). Big wheel keeps on turning: apoptosome regulation and its role in chemoresistance.. Cell Death Differ.

[54] Chen BJ, Cui X, Liu C, Chao NJ (2002). Prevention of graft-versus-host disease while preserving graft-versus-leukemia effect after selective depletion of host-reactive T cells by photodynamic cell purging process.. Blood.

[55] Thurston G, Murphy TJ, Baluk P, Lindsey JR, McDonald DM (1998). Angiogenesis in mice with chronic airway inflammation: strain-dependent differences.. Am J Pathol.

